# Clinically meaningful classes of financial toxicity for patients with diabetes

**DOI:** 10.1186/s41687-024-00834-5

**Published:** 2025-01-06

**Authors:** Minal R. Patel, Jonathan P. Troost, Michele Heisler, Noelle E. Carlozzi

**Affiliations:** 1 Department of Health Behavior and Health Equity, University of Michigan, 1415 Washington Heights, SPH 1, Room 3810, Ann Arbor, MI 48109 USA; 2 Michigan Institute for Clinical Health Research, University of Michigan, 1600 Huron Pkwy, Ann Arbor, MI 48105 USA; 3 Department of Internal Medicine, University of Michigan, 1500 East Medical Center Drive, Ann Arbor, MI 48109 USA; 4 Department of Physical Medicine and Rehabilitation, University of Michigan, 1540 E. Hospital Dr, Ann Arbor, MI 48109 USA; 5 Department of Surgery, University of Michigan, 1500 East Medical Center Dr, Ann Arbor, MI 48109 USA

**Keywords:** Financial toxicity, Diabetes, Financial burden, Clinical practice

## Abstract

**Aims:**

This study aims to improve the interpretability and clinical utility of the COmprehensive Score for financial Toxicity-Functional Assessment of Chronic Illness Therapy (COST-FACIT) by identifying distinct financial toxicity classes in adults with diabetes.

**Methods:**

Data included a sample of 600 adults with Type 1 or Type 2 diabetes and high A1c. Latent Class Analysis was used to identify subgroups of patients based on COST-FACIT score patterns.

**Results:**

We identified 3 financial toxicity classes (high, medium and low) with strong indicators of membership classification. Multiple indicators of financial stress, maladaptive cost-coping behaviors, more comorbidities, more prescribed medications, more diabetes distress, more depressive symptoms, closer to the federal poverty level, female, having lower educational attainment and being single were all significant predictors of high financial toxicity class membership. A score of 26 on the COST-FACIT was the strongest threshold for sorting high vs. medium/low financial toxicity, with a positive predictive value (PPV) of 76% and negative predictive value (NPV) of 93%.

**Conclusion:**

The COST-FACIT can be used to reliably identify people with diabetes that have high financial toxicity. Integrating this new cut-score into clinical practice may help clinical teams identify people in need of additional support due to financial toxicity.

## Introduction

Economic burden, financial stress, and cost-related adverse coping are highly relevant constructs among the 30 million Americans who have a diagnosis of diabetes [1, 2]. On average, people with diabetes have medical expenditures that are approximately twice as high as comparable individuals without diabetes [3]. The inability to engage in the numerous behaviors required to maintain optimal glycemic and other risk factor control as a result of economic burden and unmet basic needs results in complications, including high rates of avoidable acute care use, and morbidity and mortality, disproportionately among individuals with low-income [4].

Financial toxicity describes the material conditions that arise from greater expenses and lower income, the psychosocial response to those material conditions, and the coping behaviors that patients and their families adopt to manage their care, condition, and financial situation [5]. One of the most widely used instruments to measure financial toxicity is the Comprehensive Score for Financial Toxicity (COST-FACIT), a validated 11- item tool that is widely used in cancer care [6]. Our prior work demonstrated reliability and validity of the COST-FACIT among people with diabetes and high A1cs [7]. We found that worse financial toxicity was significantly correlated with higher A1C, higher levels of diabetes distress, more chronic conditions, and more depressive symptoms [7].

Financial toxicity is an important side-effect of medical treatment and disease management that should be measured and monitored in clinical practice to mitigate complications and adverse events. However, clinician’s often struggle with discerning patients at risk, and available socioeconomic indicators are not enough. Some estimates suggest that oncologists accurately identified financial distress in their patients only about 40% of the time [5]. Even when they did recognize financial hardship, they often underestimated its severity [5]. In clinical practice, screening for financial toxicity using the COST-FACIT could better flag patients with high financial toxicity for further evaluation and intervention. The COST-FACIT yields continuous values where lower scores indicate worse financial toxicity. The instrument takes less than 5 min to complete. Currently, there are limited data on thresholds of financial toxicity (e.g., high or low) measured by the COST-FACIT linked to behaviors that can lead to clinically meaningful worse health outcomes, and no data among people with diabetes. In cancer cohorts, three thresholds (no/mild, moderate, and severe financial toxicity) have been proposed with the COST-FACIT, with limited validation [8, 9]. Clinicians would benefit from a financial toxicity tool that can summarize scores into clinically distinct categories that provide actionable steps for further support.

One method for developing clinically meaningful profiles is to use a latent class analysis (LCA). LCA is a type of latent measurement model which uses observed categorical variables to assign patients into categories of an unobserved, or “latent,” variable [10, 11]. These “latent categories” are referred to as classes. An advantage of using an LCA is that it categorizes patients into discrete groups based on their common financial toxicity experiences that can be further evaluated to determine what clinical and patient characteristics are associated with each group (financial toxicity profile).

The purpose of this study is to use an LCA to define clinically distinct financial toxicity classes from the COST-FACIT among people with diabetes. We hypothesized that clinically meaningful financial toxicity classes can be identified for diabetes, particularly classes that differentiate between high and low/no financial toxicity. We also hypothesize that socioeconomic characteristics and indicators of treatment complexity will most influence sorting participants into financial toxicity classes.

## Methods

### Data source

This study is a secondary analysis of interviewer-assisted patient surveys collected as part of a larger intervention study. Data came from the baseline assessment of a randomized controlled trial that is testing approaches to addressing unmet social needs in people with diabetes and high A1c levels [12]. All study procedures were approved by the University of Michigan Institutional Review Board.

### Sample

Potential participants were identified via the University of Michigan’s Diabetes Research Registry [13] and the electronic health record through Michigan Medicine. Study participants met the following criteria: (1) 18–75 years of age; (2) diagnosis of type 1 or type 2 diabetes with prescribed oral or injectable anti-hyperglycemic medication; (3) most recent (within the past 6 months) recorded HbA1c level of ≥ 7.5% for individuals ≤ 70 years and > 8.0% for individuals between 70 and 75 years in age; (4) positive report of financial burden or cost-related non-adherence (CRN) using screening questions developed and validated from prior work [14, 15]; and (5) access to a mobile phone. Exclusion criteria included significant cognitive impairment precluding individuals from completing the study as evidenced by inability to complete study intake procedures. Individuals actively participating in another diabetes-related research study were also excluded.

Trained recruitment staff made initial contact with potential participants via telephone and screened them for inclusion/exclusion criteria. Participants who met inclusion criteria and completed informed consent for trial participation via phone prior to their baseline assessments. All participants received a monetary incentive for their participation.

A total of 6055 potential participants were initially contacted, of which 997 were confirmed to be eligible. Of those, 666 (66%) consented to participate, and 600 provided survey data. This analysis is based on 600 surveys, and 598 respondents had complete data on the COST-FACIT measure.

### Measures

In-person interviewer-assisted surveys were conducted by trained staff prior to March, 2020, when the World Health Organization declared the COVID-19 pandemic. Since then, surveys were administered over the telephone.

#### COST-FACIT

**Fig. 1:**
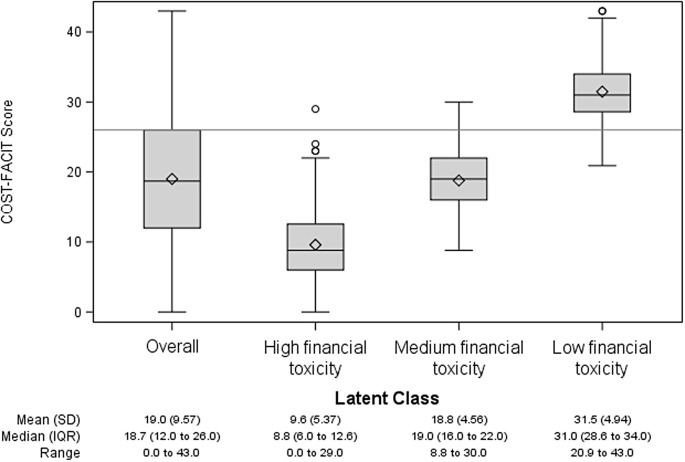
COST-FACIT score distribution for 2-class solution of high and low financial toxicity

Financial toxicity was assessed with the 11-item COST-FACIT measure (Items listed in Fig. 1) [6]. As in the original measure, each item was measured on a 5-point scale (0: not at all – 4: very much), with recall based on the past 7 days related to their diabetes management. The only changes made to the original version of the measure were to refer to their diabetes management instead of cancer treatment. A score was computed by first reverse coding 6-items, then taking the sum of all items, multiplying the sum by 11, and then dividing that number by the number of items answered. Lower scores indicated higher financial toxicity.

#### Demographic and clinic characteristics

Standard demographic data were collected, including age, gender, self-reported annual income, educational attainment, employment status, health insurance status, self-reported race/ethnicity, and marital status. Using income and household information, participants were classified using percentiles relative to the US poverty level for 2019 [16]. Other data that was collected including length of time since diagnosis with diabetes, number of chronic conditions, and type of diabetes (Type 1 or Type 2). Additional clinical measures included the 2-item validated Diabetes Distress Scale [17]. Higher scores indicated greater distress. Depressive symptoms were measured using the PHQ-4, with higher scores indicating greater severity of symptoms [18].

#### Cost-coping behaviors

We assessed cost-coping behaviors with ten individual items from validated measures and national surveys [14, 15, 19]. Specifically, participants were asked whether they engaged in any of the following behaviors during the last 12 months due to financial burden for diabetes: took less medications, skipped medication doses, delayed or decided to not fill a prescription, and delayed or decided not to see a healthcare provider (4-point Likert scale: never - often). We also asked participants how often over the past six months, they ‘borrowed money from someone’, ‘over-drafted on checking account’, ‘maxed out the limit on one or more credit cards’, or ‘did not pay bills on time’ (5-point Likert scale: never- always) [20]. These behaviors were analyzed as dichotomous variables, with ‘often’ and ‘always’ indicating ‘yes’. The percentage of participants with a positive response was reported for each of the ten items.

#### Economic burden

Economic burden was captured through four assessments related to their health: (1) self-report of out-of-pocketing spending on diabetes in the past 30 days on eight indicators (medications, physical activity membership, doctor visits, home blood testing supplies, laboratory tests, transportation for healthcare visits, physical therapy, low carb or low sugar foods). Items were assessed on a four-category scale ($0, $1–50, $51–100, over $100); (2) self-report of average, monthly out-of-pocket spending to manage all health conditions. Items were assessed on an 11-point Likert scale in $50 increments between $0-501; (3) self-report of areas of needed assistance. Items were adapted from: the Accountable Health Communities Health-Related Social Needs Screening Tool, the Health Leads Social Needs Screening Toolkit, and the Kaiser Permanente Your Current Life Situation Questionnaire [21, 22]. Items assessed the presence of everyday needs over the past 12 months such as food, housing, energy/utilities; (4) four items adapted from the Financial Management Behavior Scale (FMBS) [20]: self-report of whether over the past 6 months, participants maxed out limit on one or more credit cards, borrowed money from someone, or over-drafted on checking account. Items were assessed on a 5-point Likert Scale (1-never to 5 always).

#### Financial stress attributed to diabetes

Financial stress attributed to diabetes was captured through self-report of aspects of diabetes management endorsed as financially burdensome (e.g., health insurance, buying healthy food, medications, testing supplies, physical activity) assessed on a 5-point Likert scale (not at all – very much).

### Statistical analysis

All analyses were conducted using SAS 9.4 (SAS Institute Inc., 2014). One item on the COST-FACIT measure had a high number of ‘not applicable’ responses- ‘I am concerned about keeping my job’. Sensitivity analyses were run on both the full measure (scoring of all 11 items), as well as after eliminating the item with low response rates. Given that the analyses did not differ based on inclusion of the removed item, we report on the 10-item version (given the larger number of observations with this approach).

#### LCA class selection

LCA [10, 11] is a posterior membership probability modeling technique used to identify latent (i.e., “unobserved”) categorical subgroups of respondents based on their responses to categorical questions—in this study we created latent classes based on their responses to economic burden, cost-coping, financial stress variables, and depressive symptoms and diabetes distress. Analyses were performed using PROC LCA in SAS 9v.4 [23]. When performing an LCA, the user specifies the number of classes (and then the algorithm identifies a class structure and assigns class membership probabilities to each participant). First, the model number of classes is determined by iteratively running a series of models with an increasing number of classes (e.g., 2-class solution, 3-class solution, 4-class solution etc.). The optimal number of classes was determined based on both model fit (AIC, BIC, and entropy) and model interpretability for practical application. Once class number was determined, respondents were classified into latent classes based on maximum posterior probability.

LCA requires a minimum of 200 subjects to guarantee stability of model estimates [24, 25]. Our sample was well above that threshold.

#### Predictors of class membership

Participants were assigned latent classes based on highest posterior probabilities of class membership. Predictors of class membership (high vs. low) were tested using chis-square tests for categorical variables and Kruskal-Wallis tests for continuous variables.

We used regression models and Receiver Operator Characteristic (ROC) curves to identify the strongest thresholds of COST-FACIT scores in predicting high vs. low financial toxicity class membership (based on highest combined value of positive and negative predictive values). Sensitivity analyses were conducted for people with Type 1 and Type 2 diabetes. No differences in class membership thresholds were observed by diabetes type.

## Results

### Study population

A total of 600 participants were included in the analytic sample (Table 1). On average, participants were 53 years of age (standard deviation or SD = 13), 56% (*n* = 332) were female, 35% (*n* = 210) reported non-white race, and 87% (*n* = 520) reported some college education or above. 12% (*n* = 73) were classified as living on incomes less than 100% of the poverty level, 16% (*n* = 94) 100–200% of the poverty level, 44% (*n* = 261) 201–400% of the poverty level, and 28% (*n* = 163) 401% or more above the poverty level. 98% (*n* = 587) reported having either public or private health insurance. Mean years living with diabetes was 17 (SD = 11). Participants on average were managing a mean number of 4 chronic conditions (SD = 2.3), and 46% (*n* = 272) reported taking 7 or more medications. 28% (*n* = 165) reported moderate-severe depressive symptoms, and 63% (*n* = 377) reported high diabetes-related distress (Table 2).

**Table 1:** Sample demographic and clinical characteristics (*n* = 600)

Characteristic	Overall *n* (%)
Age (Mean (SD))	53.5 (13.00)
Federal Poverty Level Categories	
Less than 100 FPL	73 (12)
100–200% of FPL	94 (16)
201–400% of FPL	261 (44)
Greater than 400% of FPL	163 (28)
Gender/Sex	
Female	332 (56)
Male	264 (44)
Other	2 (0)
Race Recode	
Non-Hispanic White	386 (65)
Non-Hispanic Black or African American	101 (17)
Hispanic	31 (5)
Asian	23 (4)
Multiple Race	42 (7)
Other	13 (2)
Education	
Less than high school	9 (2)
High school graduate or GED equivalent	71 (12)
Some college/or associate degree	269 (45)
College graduate	249 (42)
Health Insurance (% yes)	587 (98)
Marital Status: Married or partnered, *n* (% yes)	339 (57)
Diabetes Diagnosis, *n* (%)	
Type 1	129 (22)
Type 2	469 (78)
Mean years living with diabetes (Mean (SD))	17.4 (11.17)
Comorbidities (Mean (SD))	4.2 (2.37)
Number of medications prescribed across all conditions	
1–2	51 (9)
3–4	112 (19)
5–6	162 (27)
7 or more	272 (46)

**Table 2:** Comparison of high and low financial toxicity latent classes

Characteristic		Overall *n* (%)	High financial toxicity *n* (%)	Medium financial toxicity *n* (%)	Low financial toxicity *n* (%)	*p*-value (overall)	*p*-value (high vs. medium/low)
**Demographic factors**							
Age (Mean (SD))		53.5 (13.00)	52.9 (11.25)	52.3 (14.11)	56.2 (12.80)	0.004	0.08
Federal Poverty Level Categories						< 0.001	< 0.001
Less than 100 FPL		73 (12)	48 (25)	24 (10)	1 (1)		
100–200% of FPL		94 (16)	49 (26)	36 (14)	9 (6)		
201–400% of FPL		261 (44)	75 (39)	129 (51)	57 (38)		
Greater than 400% of FPL		163 (28)	18 (9)	63 (25)	82 (55)		
Gender/Sex						< 0.001	< 0.001
Female		332 (56)	131 (68)	142 (55)	59 (40)		
Male		264 (44)	61 (32)	115 (45)	88 (59)		
Other		2 (0)	0 (0)	0 (0)	2 (1)		
Race						0.001	0.001
Non-Hispanic White		386 (65)	118 (62)	159 (62)	109 (73)		
Non-Hispanic Black or African American		101 (17)	49 (26)	39 (15)	13 (9)		
Hispanic		31 (5)	5 (3)	17 (7)	9 (6)		
Asian		23 (4)	4 (2)	15 (6)	4 (3)		
Multiple Race		42 (7)	13 (7)	17 (7)	12 (8)		
Other		13 (2)	2 (1)	9 (4)	2 (1)		
Education						0.05	0.04
Less than high school		9 (2)	4 (2)	3 (1)	2 (1)		
High school graduate or GED equivalent		71 (12)	30 (16)	32 (12)	9 (6)		
Some college/or associate degree		269 (45)	92 (48)	113 (44)	64 (43)		
College graduate		249 (42)	66 (34)	109 (42)	74 (50)		
						0.46	0.76
Health Insurance (% yes)		587 (98)	188 (98)	251 (98)	148 (99)		
Marital Status: Married or partnered		339 (57)	84 (44)	153 (60)	102 (68)	< 0.001	< 0.001
**Clinical factors**							
Diabetes Diagnosis						0.32	0.35
Type 1		129 (22)	37 (19)	63 (25)	29 (19)		
Type 2		469 (78)	155 (81)	194 (75)	120 (81)		
Comorbidities (Mean (SD))		4.2 (2.37)	5.2 (2.13)	3.8 (2.36)	3.6 (2.27)	< 0.001	< 0.001
Mean years living with diabetes (Mean (SD))		17.4 (11.17)	17.2 (11.79)	17.3 (10.43)	17.6 (11.67)	0.77	0.48
Number of medications prescribed across all conditions						< 0.001	< 0.001
1–2		51 (9)	6 (3)	29 (11)	16 (11)		
3–4		112 (19)	27 (14)	56 (22)	29 (19)		
5–6		162 (27)	38 (20)	75 (29)	49 (33)		
7 or more		272 (46)	120 (63)	97 (38)	55 (37)		
Diabetes-related Distress						< 0.001	< 0.001
No distress		150 (25)	34 (18)	49 (19)	67 (45)		
Moderate distress		71 (12)	22 (11)	27 (11)	22 (15)		
High distress		376 (63)	136 (71)	180 (70)	60 (40)		
Depressive Symptoms						< 0.001	0.01
None		259 (43)	41 (21)	112 (44)	106 (71)		
Mild		174 (29)	62 (32)	78 (30)	34 (23)		
Moderate		94 (16)	43 (22)	43 (17)	8 (5)		
Severe		70 (12)	46 (24)	23 (9)	1 (1)		
**Behaviors**							
**Cost-related Non-Adherence Sometimes/Often in past 12 months**							
Taken Smaller Doses of Diabetes Medicine to Make Last Longer		97 (16)	48 (25)	43 (17)	6 (4)	< 0.001	< 0.001
Skipped Doses of Diabetes Medicine to Make Last Longer		85 (14)	38 (20)	42 (16)	5 (3)	< 0.001	0.01
Delayed Getting Diabetes Prescriptions Filled Because of Cost		147 (25)	62 (32)	74 (29)	11 (7)	< 0.001	0.003
Decided Not to Fill Prescription for Diabetes Medicine Because of Cost		104 (17)	50 (26)	41 (16)	13 (9)	< 0.001	< 0.001
Delayed Seeing Healthcare Provider for Diabetes Due to Cost		112 (19)	56 (29)	48 (19)	8 (5)	< 0.001	< 0.001
Did Not See Healthcare Provider for Diabetes Due to Cost		68 (11)	36 (19)	28 (11)	4 (3)	< 0.001	< 0.001
**Financial Behaviors in Past 6 Months- Often/Always**							
Maxed Out Limit On One or More Credit Cards		66 (13)	40 (28)	21 (9)	5 (4)	< 0.001	< 0.001
Borrowed Money From Someone		44 (7)	33 (17)	11 (4)	0 (0)	< 0.001	< 0.001
Over-Drafted on Checking		25 (4)	18 (10)	5 (2)	2 (1)	< 0.001	< 0.001
**Unmet Basic Needs in Past 12 Months- Often True**							
Food You Bought Did Not Last and Did Not Have Money to Get More		50 (8)	41 (21)	9 (4)	0 (0)	< 0.001	< 0.001
Electric, Gas, Oil or Water-Company Threatened to Shut Off Services in Home						< 0.001	< 0.001
Yes		122 (20)	68 (36)	49 (19)	5 (3)		
Already shut off		1 (0)	1 (1)	0 (0)	0 (0)		
**Financial stress**							
**Unmet Basic Needs in Past 12 Months- Often True**							
Worried Food Would Run Out Before Got Money To Buy More		76 (13)	65 (34)	11 (4)	0 (0)	< 0.001	< 0.001
Worried About Not Being Able to Pay Rent, Mortgage or Other Housing Costs		78 (13)	64 (33)	14 (5)	0 (0)	< 0.001	< 0.001
**Perceptions of Financial Burden with Aspects of Diabetes Management- Quite a Bit/Very Much**							
Medications		219 (37)	109 (57)	95 (37)	15 (10)	< 0.001	< 0.001
Testing Supplies		175 (30)	72 (38)	77 (30)	26 (18)	< 0.001	< 0.001
Buying Healthy Foods		248 (42)	124 (66)	101 (39)	23 (15)	< 0.001	< 0.001
Physical Activity Memberships		104 (25)	48 (37)	47 (24)	9 (9)	< 0.001	< 0.001
Nutrition Counseling		73 (17)	33 (23)	32 (18)	8 (8)	0.01	0.05
Diabetes Education		48 (10)	26 (17)	16 (8)	6 (5)	0.01	0.002
Diabetes Management Programs		52 (13)	29 (22)	18 (10)	5 (5)	< 0.001	< 0.001
Healthcare Visits		113 (19)	64 (34)	43 (17)	6 (4)	< 0.001	< 0.001
Health Insurance		264 (45)	104 (56)	127 (50)	33 (22)	< 0.001	< 0.001
Transportation Related to Healthcare		91 (16)	59 (32)	29 (12)	3 (2)	< 0.001	< 0.001
**Out-of-pocket expenses**							
**Amount Spent Out-Of-Pocket in Past 30 Days- $0–25**							
All Medications Recommended by Provider		194 (33)	71 (38)	72 (28)	51 (35)	0.58	0.39
Doctor Visits		328 (56)	116 (61)	124 (50)	88 (60)	0.07	0.23
Laboratory Tests		455 (81)	140 (77)	192 (79)	123 (84)	0.36	0.14
Home Blood Testing Supplies		377 (63)	122 (64)	150 (59)	105 (71)	0.06	0.68
Physical or Occupational Therapy		563 (96)	181 (96)	238 (94)	144 (97)	0.24	0.72
Home Healthcare		580 (97)	184 (96)	249 (98)	147 (99)	0.39	0.27
Special Low Carb and Low Sugar Foods		263 (46)	66 (37)	113 (45)	84 (58)	0.002	0.005
Memberships for Physical Activity		512 (86)	170 (89)	207 (81)	135 (91)	0.09	0.26
Transportation for Healthcare Visits		393 (66)	95 (50)	176 (68)	122 (82)	< 0.001	< 0.001
**Amount Spent Out-Of-Pocket to Manage All Health Conditions During Average Month**							
$501 or more		54 (9)	19 (10)	19 (8)	16 (11)	0.02	0.29
**Cost-facit items**							
Know Have Enough Money in Savings, Retirement or Assets to Cover Costs of Treatment		2.5 (1.43)	1.6 (1.12)	2.3 (1.14)	3.9 (1.18)	< 0.001	
Out-Of-Pocket Medical Expenses Are More Than Thought		2.9 (1.36)	2.6 (1.46)	2.9 (1.20)	3.4 (1.36)	< 0.001	
Worry of Financial Problems in Future As Result of Illness or Treatment		2.3 (1.27)	1.7 (1.14)	2.1 (0.98)	3.4 (1.21)	< 0.001	
Feel Have No Choice About Amount of Money Spent on Care		2.2 (1.25)	1.9 (1.17)	2.1 (1.03)	2.7 (1.53)	< 0.001	< 0.001
Frustrated Cannot Work or Contribute As Much As Usual		2.9 (1.61)	1.6 (0.98)	3.2 (1.49)	4.1 (1.31)	< 0.001	< 0.001
Satisfied With Current Financial Situation		2.2 (1.27)	1.1 (0.58)	2.1 (0.92)	3.6 (1.01)	< 0.001	< 0.001
Able to Meet Monthly Expenses		3.3 (1.25)	2.3 (1.07)	3.4 (0.88)	4.6 (0.56)	< 0.001	< 0.001
Feel Financially Stressed		2.7 (1.37)	1.4 (0.79)	2.7 (0.98)	4.3 (0.72)	< 0.001	< 0.001
Concerned About Keeping Job and Income		3.4 (1.55)	2.5 (1.65)	3.3 (1.39)	4.4 (1.00)	< 0.001	< 0.001
Diabetes Expenses Reduced Satisfaction with Present Financial Situation		3.3 (1.38)	2.5 (1.40)	3.3 (1.16)	4.3 (0.99)	< 0.001	< 0.001
Feel in Control of Financial Situation		2.6 (1.25)	1.7 (1.07)	2.6 (0.68)	4.0 (0.97)	< 0.001	< 0.001
COST-FACIT Score		19.0 (9.57)	9.6 (5.37)	18.8 (4.56)	31.5 (4.94)	< 0.001	< 0.001

### Latent class selection

**Fig. 2:**
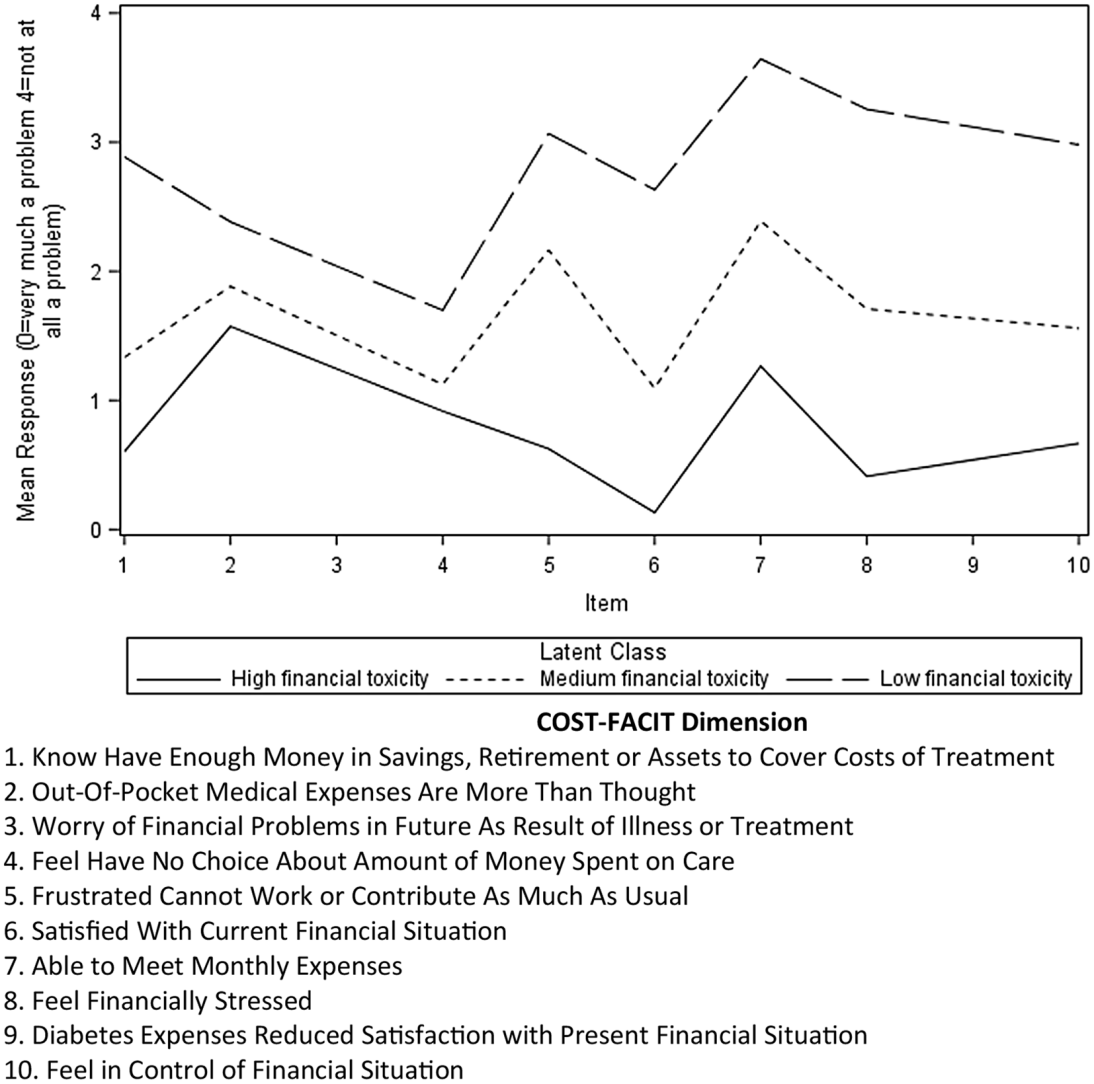
Mean responses by COST-FACIT scale item for high and low financial toxicity latent classes

Model selection indices were computed for 2 class (AIC: 9295.67; BIC: 9651.83; Entropy: 0.88), 3 class (AIC: 8854.85; BIC: 9391.28; Entropy: 0.86), and 4 class (AIC: 8724.19; BIC: 9440.89; Entropy: 0.87) latent models. A three-class model was favored for clinical interpretability and utility, and parsimony in the absence of evidence for a need to use a more complex model (Fig. 1). Figure 2 shows mean response by COST-FACIT items. The three-class model sorted participants into three clear classes: high financial toxicity, medium financial toxicity and low financial toxicity. Lower COST-FACIT scores mean worse financial toxicity. Mean COST-FACIT scores for the high financial toxicity class was 9.6 (SD = 5.37), 18.8 (SD = 4.56) for medium financial toxicity, and 31.5 (SD = 4.94) for the low financial toxicity class.

### Comparison of latent classes by participant factors

In comparing high versus medium/low financial toxicity classes (Table 2), several significant differences were observed. Individuals in the high financial toxicity class were more likely to be closer to the federal poverty level, female, have lower educational attainment, and be unmarried or unpartnered.

Clinically, this group had a higher number of comorbidities, were more frequently prescribed seven or more medications, experienced greater diabetes-related distress, and reported more severe depressive symptoms.

Behaviorally, they were more likely to engage in cost-related non-adherence, such as skipping or delaying medication and healthcare visits due to cost, and reported higher incidences of maladaptive financial behaviors, including overdrafting accounts, borrowing money, and maxing out credit cards. They also faced more unmet basic needs, such as food insecurity and utility shut-offs, and reported greater financial stress attributed to various aspects of diabetes management, including healthcare expenses, medications, and buying healthy food.

Out-of-pocket spending differed, with those in the high financial toxicity class being less likely to report spending within the lowest range for items like transportation and special low-carb foods.

### Predictive models

**Fig. 3:**
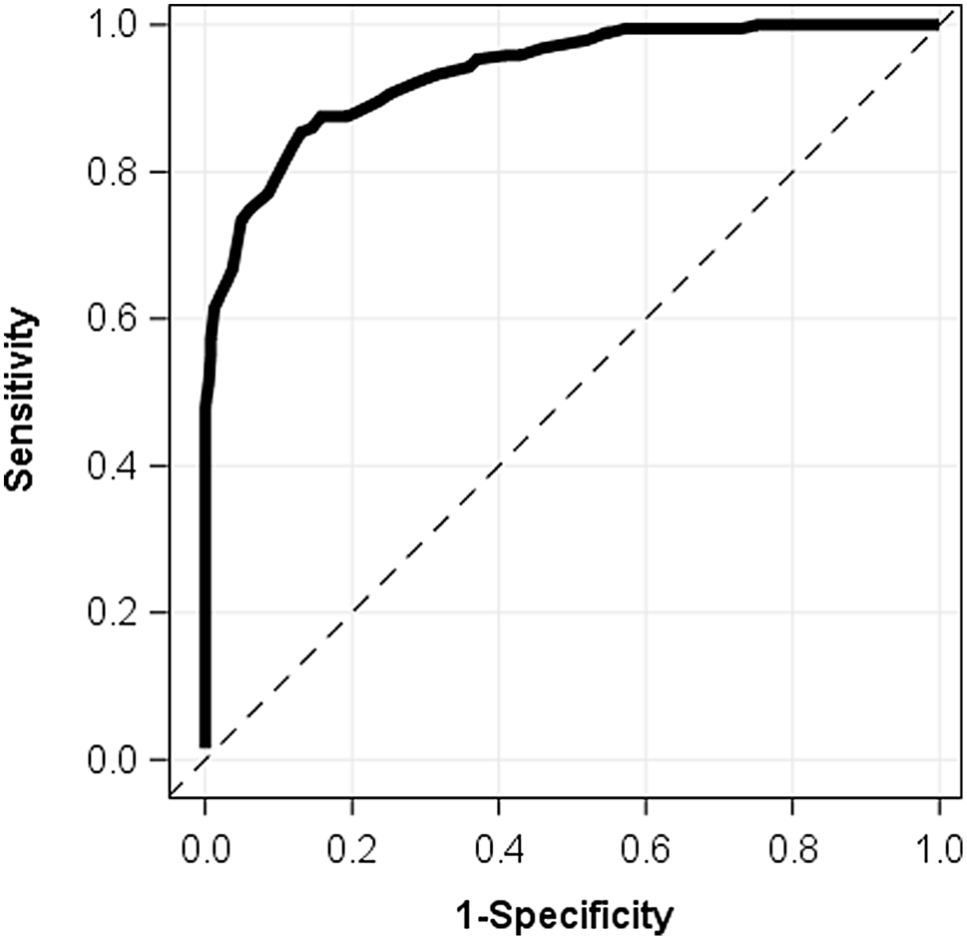
Receiver Operator Characteristic (ROC) curves for models of COST-FACIT scores predicting high vs. medium/low financial toxicity

Figure 3 shows ROC curves based on the 3-class solution determined from the latent class analysis. A score of 26 is the strongest threshold at sorting high vs. medium/low financial toxicity. This threshold has a PPV of 76% and NPV of 93%.

## Discussion

Validated measures, such as the COST-FACIT, for detecting financial toxicity as a side-effect of diabetes treatment and management provide significant promise for researchers and clinicians. However, adoption of the COST-FACIT into clinical practice requires the ability to discern clinically meaningful categories of financial toxicity that assist providers in identifying who requires further attention. Using Latent Class Analysis to identify subgroups of patients based on COST-FACIT score patterns, we identified three financial toxicity classes (high, medium and low) with strong predictors of membership classification. These subgroups were predicted by several demographic, clinical and behavioral characteristics.

This is the first study to establish a threshold for classification of financial toxicity among people with diabetes. Within our study, we were able to establish a score of 26as the strongest threshold for sorting high and medium/low financial toxicity. Our findings compare to other studies among people with cancer. In one study of patients with gynecologic cancers, financial toxicity was determined by a COST-FACIT score < 26 [8]. In another preliminary study of two types of cancer patients, a grading system was developed for the COST-FACIT by the initial proposers of the tool that also supported a financial toxicity threshold of < 26 COST-FACIT score [9].

Our study also confirmed risk factors for high financial toxicity described more broadly in cancer and other chronic disease populations [26–29]. These include factors that may contribute to a lower socioeconomic position such as federal poverty level, female, lower educational attainment, and not married [26–29]. They also included clinical factors that would lead to greater out-of-pocket healthcare expenses such as being prescribed more medications and having more comorbidities [26–29]. Our study went further than prior studies and also found maladaptive coping and financial behaviors, and unmet social needs as predictors of high financial toxicity.

There are limitations to this study that should be noted. The sample in this study consisted of adults with diabetes, high HbA1cs, and self-reported indication of unmet social determinants of health who had been recruited to an intervention study in one health system. We did not have information on possible levels of financial toxicity among those who chose not to participate, or did not meet other eligibility criteria for the trial. This therefore limits the generalizability of our findings. Part of our sample was also recruited prior to the COVID-19 pandemic which exacerbated socioeconomic inequities, however, we did not observe significant differences in financial toxicity between our pre-pandemic and post-pandemic recruits in our sample. We did not query participants about financial toxicity related to comorbidities, although this would be an important area of further exploration. Although our sample was heterogeneous in terms of race/ethnicity, further work is warranted to assess the COST-FACIT across people with diabetes and varying levels of glycemic control and reported unmet social needs to confirm the stability of the latent classes found in this study. It also would be important to further test briefer versions of the COST scale for screening and intervention in clinical practice.

Despite these limitations, the findings provide important implications for clinical practice. The practice of stratifying patients into clinically actionable groups is common in the clinical workflow across a range of health conditions. This study of latent classes for financial toxicity among people with diabetes will provide a much-needed patient-reported indicator with clinical utility of the significant economic burden that a wide range of patients are facing with managing diabetes over the long-term [1, 7]. We envision that our score for sorting high and medium/low financial toxicity among people with diabetes will better optimize clinic resources to assist patients in navigating low-cost options for their care, and in turn better identify patients who may need more support. This can include more precise criteria for referrals to financial counseling or social work services, which may in turn lead to lower cost medication or treatment options, and support with other unmet social needs that contribute to financial toxicity.

## Conclusion

The COST-FACIT can be used to reliably identify people with diabetes that are at risk for high financial toxicity. Integrating this new cut-score into clinical practice may help clinical teams identify people in need of additional support due to financial toxicity, as well as to provide treatment recommendations that may help mitigate (or at least not exacerbate) the financial burden of these patients.

## Data Availability

The data sets used during this study are not publicly available because it was not required at the time of the initial grant award, and as such, participants did not consent to future use of their data. Sharing the data publicly would require re-consenting and increase the risk of breach of confidentiality.
